# Uncorrected refractive errors, visual impairment and need for spectacles among children and adolescents in eastern, China

**DOI:** 10.1371/journal.pone.0332142

**Published:** 2025-09-16

**Authors:** Yue Zhou, Qi Cai, Xiaojuan Chen, Xiaobo Huang, Zhimin Sun, Yu Song, Lele Li, Yan Zhu, Wang Yong, Peirong Lu

**Affiliations:** 1 Department of Ophthalmology, Second Affiliated Hospital of Nantong University, Nantong, Jiangsu Province, China; 2 Department of Ophthalmology, The First Affiliated Hospital of Soochow University, Suzhou, Jiangsu Province, China; The Ohio State University, UNITED STATES OF AMERICA

## Abstract

**Background:**

Uncorrected refractive errors (URE) are the leading preventable cause of visual impairment (VI) in children globally, with China facing a critical dual challenge of high myopia prevalence and insufficient spectacle coverage among youth. Despite eastern China’s rapid development, population-based data on URE, VI, and need for spectacles remain scarce, particularly regarding the understudied role of anisometropia and subtype-specific refractive risks. This study evaluates these unmet needs to inform targeted interventions.

**Methods:**

A cross-sectional school-based study was conducted in Nantong, China, including participants 7–19 years of age. All participants underwent assessments of their uncorrected visual acuity, presenting visual acuity (PVA), and best-corrected visual acuity. URE was defined as PVA worse than 0.3 logMAR (6/12 Snellen) with ≥1 line improvement (≥0.1 logMAR) after correction in either eye.VI was defined as PVA < 6/12 in the better eye. Need for spectacles was defined as the total prevalence of refractive error requiring correction, including unmet, under-met, and met needs. Non-cycloplegic autorefraction was assessed for each participant.

**Results:**

Of the 9,864 participants, 9,438 were included in the analysis. The total prevalence of URE, VI and need for spectacles was 15.7% (95% CI: 15.0–16.5; n = 1,485),4.9% (95% CI: 4.9–5.3; n = 459) and 55.9% (95% CI: 54.9–56.9; n = 5,275), respectively. Multivariate analysis showed that factors such as female sex (aOR: 1.24, 95% CI: 1.09–1.40), wearing spectacles (aOR: 0.16, 95% CI: 0.14–0.19), older age groups (e.g., aOR: 3.92 for 13–14 years), hyperopia (aOR: 13.08, 95% CI: 7.67–22.31), myopia (aOR: 18.65, 95% CI: 12.54–27.77), and anisometropia (aOR: 1.87, 95% CI: 1.64–2.12) were associated with URE. For VI, significant associations included female sex (aOR: 1.20, 95% CI: 0.98–1.47), hyperopia (aOR: 7.23, 95% CI: 1.60–32.61), myopia (aOR: 53.04, 95% CI: 19.68–142.95), and rural residence (aOR: 1.53, 95% CI: 1.25–1.87). Factors such as older age (highest aOR: 11.77 for 19 years), female sex (aOR: 1.58, 95% CI: 1.42–1.77), hyperopia (aOR: 16.56, 95% CI: 10.97–25.01), myopia (aOR: 28.88, 95% CI: 21.83–38.19), astigmatism (aOR: 2.50, 95% CI: 2.22–2.82), and anisometropia (aOR: 1.37, 95% CI: 1.21–1.55) were associated with need for spectacles.

**Conclusion:**

Although the prevalence of VI among children and adolescents in eastern China was low, the prevalence of URE and the need for spectacles were high. Myopia was the most important risk factor for URE, VI, and need for spectacles, and the impact of anisometropia on URE, VI, and need for spectacles cannot be ignored. Further research on adjusting intervention strategies is needed to eliminate preventable visual impairments.

## Introduction

Uncorrected refractive errors (URE) are the leading cause of global avoidable visual impairment (VI) [1]. It accounts for moderate to severe VI worldwide and is the second most common cause of blindness [2]. In China, recent epidemiological studies indicate a significant rise in URE prevalence among children aged 6–12 years, with early-onset myopia (≤8 years) increasing by 23% between 2015 and 2021 [3]. The World Health Organization has estimated that approximately 12.8 million school-aged children (ages: 5–15 years) are visually impaired due to URE, with half living in China [4]. VI was one of the most serious disabilities affecting over a billion people worldwide [5]. For school-aged children and adolescents, URE and VI tends to impede effective learning, cause quality of life issues and self-esteem issues, and are associated with developmental disorders [6–8].

Refractive errors are mainly divided into three types: myopia, hyperopia, and astigmatism [9]. Anisometropia refers to a unique refractive condition in which the refractive error differs by ≥1.00 D between the two eyes of an individual [10], it is not only considered a contributing factor to amblyopia, but can also lead to binocular visual dysfunction, particularly stereopsis impairment [11]. Refractive errors and anisometropia can be corrected with spectacles; timely diagnosis prevents negative impacts of URE [12].

Previous studies [13–15] have primarily focused on estimating URE prevalence based on isolated refractive error types without concurrently analyzing interactions between coexisting refractive errors [16] (such as the association between astigmatism and myopia progression), the synergistic effects of refractive errors and anisometropia [17], or differential visual outcomes across refractive error subtypes [9,18,19]. Harrington et al.[20] comprehensively considered various types of refractive errors in their study of VI in Irish children, but did not consider the factor of anisometropia. These gaps suggest that the combined influence of multiple refractive error types and anisometropia on URE and VI remains underexplored in population-based studies.

Due to the increased academic demand, children and adolescents in eastern China have a high prevalence of myopia and VI [21,22]. However, population-based data on URE, need for spectacles, Refractive Error Coverage (REC) and Effective Refractive Error Coverage (eREC) in Chinese children and adolescents’ population are limited. In the present study, we aimed to report the prevalence of URE, VI and need for spectacles among individuals aged 7–19 years in eastern China. The second objective was to evaluate the independent effects of different refractive error types (myopia, hyperopia, astigmatism) and anisometropia on URE, VI, and need for spectacles.

## Methods

### Study design

The Nantong school-aged children Eye Study (NSES) was a school-based study of eye conditions in Jiangsu Nantong, a moderately sized city of 7.749 million people living on the east of China. Nantong city was characterized by a subtropical maritime monsoon climate (average 15.0 ºC yearly), lower elevation (around 2–6.5 meters), and plains, with a relatively stable demographic structure. In 2022, the average annual disposable income of residents in Jiangsu Province was 49,862 RMB (approximately USD 6,868), and the average annual disposable income of residents in Nantong City was 49,093 RMB (approximately USD 6,762) [20].

The NSES is mainly designed to investigate the prevalence and associated factors of refractive errors and refractive correction in school-age children in Nantong China and was registered on Chinese Clinical Trial Registry (http://www.chictr.org. cn, ChiCTR2300077367). This school-based cross-sectional study was conducted from August to September 2023.The study was approved by the ethics committee of the Second Affiliated Hospital of Nantong University, China (approval number: 2023KT122). All protocols used in this study followed the tenets of the Declaration of Helsinki [23]. Participants under the age of 18 should obtain written informed consent from at least one guardian, and participants aged 18 or older should directly obtain written informed consent. This study followed the Strengthening of the Reporting of Observational Studies in Epidemiology (STROBE) reporting guidelines. While the NSES is a longitudinal cohort, the present analysis utilizes cross-sectional data from the baseline survey to estimate prevalence and associated factors. Longitudinal outcomes will be reported in future studies.

### Sample selection

A school-based study conducted in Lhasa showed that the prevalence of URE was 11.70% in 2021 [15]. To achieve a power of 80%, the sample size was calculated using the formula [24,25], n = t^2^pq/d^2^, assuming a design effect of 1.5 due to cluster sampling and a nonresponse rate of 5% (t = 2 for a 95% confidence interval (CI), q = 1-P, d = 0.1 P). The total sample size was at least 3,019. To ensure better multifactor analyses, more samples were included in the protocol. A stratified cluster sampling method was used. According to the 2023 Nantong Municipal Education Statistical Report, the sampling frame included 327 primary schools, 214 junior high schools, and 97 senior high schools. Thirty schools (10 from each educational stage) were randomly selected using a computer-generated list stratified by urban/rural location. Within each selected school, two classes per grade were randomly chosen using a computer-generated random number table. All students in the selected classes were invited to participate. Participants who did not cooperate with the examination had missing data, or provided incorrect information were excluded from the analysis. The inspection site was set in each school. Before the study began, researchers standardized the lighting conditions at each inspection site using digital lux meters (Model TES-1336A, Shenzhen), ensuring an illumination of 300–500 lux as per the Chinese National Standard for Visual Acuity Charts (GB 11533−2011). Autorefractors were calibrated daily using manufacturer-provided test lenses. The proportion of children and adolescents who agreed to participate was 98.5%.

### Eye examination procedure

All technicians participate in a series of unified and centralized vision acuity (VA) testing and automatic refraction practice training supported by NSES. Only technicians who pass the post-training assessment can be considered qualified screening personnel. An experienced clinical team comprising five certified optometrists and five ophthalmologists from the Second Affiliated Hospital of Nantong University performed examinations. All technicians received standardized training and passed post-training assessments. Demographic data was collected before examination including age, gender, education level. Before the test, participants were asked and recorded whether they wore spectacles, contact lenses, or orthokeratology lenses. Daily spectacle wearers were asked to bring their glasses to the examination site. Because orthokeratology produces overnight corneal reshaping with daytime unaided correction, orthokeratology users were treated as spectacle wearers [26]. Given that residual corneal changes can persist beyond 24 hours after discontinuation, we did not use same-day non-cycloplegic autorefraction from orthokeratology users to assign refractive subtypes [27,28]; instead, pre-treatment clinical records (routine pre-fitting documentation including unaided VA and baseline non-cycloplegic refraction) were obtained from the fitting clinics or the affiliated hospital and were used only for refractive-subtype classification [29]. Presenting VA for orthokeratology users was measured under the same standardized conditions as for other participants (logMAR chart, fixed distance and illumination). Users of other contact lenses removed lenses after the PVA test; subsequent refraction followed the same protocol as for non–contact-lens wearers.

All participants received standardized ophthalmic examination and non-cycloplegic autorefraction was measured using three repeated measurements via an autorefractor (WSRMK-8000; Biobase, Shandong, China). Manual streak retinoscopy was conducted if autorefractor measurements varied by >0.50 D between two readings. If any two measurements differed by >0.50 D, three additional autorefractor measurements were performed, followed by retinoscopy or subjective refraction by senior optometrists to confirm the final value. Unaided visual acuity, presenting visual acuity (PVA), and best-corrected visual acuity (BCVA) were measured using a logMAR-based liquid crystal tumbling E chart (WSVC-100; Qingdao Optometry, China) at 5 meters under standardized illumination (300 cd/m²). VA was recorded as the smallest line where ≥50% of optotypes were correctly identified, following the Chinese national standard (GB 11533−2011). The chart followed the logarithmic progression of the Early Treatment Diabetic Retinopathy Study (ETDRS) design, where each line represents a 0.1 logMAR increment (e.g., 0.0, 0.1, 0.2 logMAR). Participants were required to correctly identify at least three out of five optotypes (60%) to pass a line. Snellen equivalents were derived from logMAR values (e.g., 0.3 logMAR = 6/12 Snellen). URE was defined as PVA worse than 0.3 logMAR (6/12 Snellen) in either eye, with improvement of ≥0.1 logMAR (≥1 line) after correction. PVA was defined by the detected visual acuity when tested wearing currently available refractive correction, if any [30]. Participants who used orthokeratology lenses were not specifically instructed to stop or continue using them. Participants who used other contact lenses were instructed to remove them for further refraction after the PVA examination. BCVA was measured after subjective refraction based on autorefractor results. Experienced optometrists refined the spherical, cylindrical, and axis components using a trial frame and lenses until participants achieved their maximum visual acuity, ensuring BCVA reflected the participant’s optimal visual potential. Participants with amblyopia and low vision were transferred to the Second Affiliated Hospital of Nantong University for further investigation.

### Anthropometry

Conventional physical examinations including weight and height were conducted by well-trained health workers. The World Health Organization has described a measurement scheme for height and weight [31]. The weight parameter was measured using a beam-scale to the nearest 0.1 kg, without wearing thick clothes, while wearing minimum clothing, and keeping the body steady. The height parameter was precise to 0.1 cm without shoes, with back to the column and the torso naturally straight, head upright, and eyes looking straight ahead. BMI was then calculated as weight (kg)/height (m)^2^.

### Definitions of visual-acuity outcomes and refractive-error classifications

URE was defined as presenting visual acuity (PVA) <6/12 in either eye (regardless of spectacle use), improving by ≥1 line with BCVA refraction. This definition encompasses both individuals without spectacles and those with inadequately corrected refractive errors (e.g., outdated or incorrect prescriptions that fail to achieve PVA ≥ 6/12). Our definition of URE was based on the validated cutoffs for refractive correction in the study of refractive error in children [32–34], and the fact that imbalanced input of binocular visual signals may lead to myopia progression, stereoscopic visual dysfunction, and affect adult stereoscopic visual acuity [35]. VI was defined as PVA < 6/12 in the better eye. This definition was in line with the following WHO categories: Mild VI defined as uncorrected or presenting VA (spectacle-corrected VA, if worn) worse than 6/12 [36]. VA was recorded as the log MAR value corresponding to the smallest line with ≥50% correct responses, consistent with WHO recommendations [36]. To minimize the potential impact of spurious associations between anisometropia and ametropia, subjects were categorized according to the SE in less ametropic eyes (less positive or more negative refractive error) [17,37]. Spherical equivalent refraction (SE) was calculated as follows: SE = negative cylindrical degree × 0.5 + spherical degree. Myopia was defined as SE ≤ −0.5 D. Emmetropia was defined as −0.5 D < SE ≤ +0.5 D. Hyperopia was defined as an SE > +0.5 D. Anisometropia was defined as the absolute SE difference ≥ 1.0 D between eyes. Astigmatism was defined as cylinder power 1.0 diopters (D) or greater [38,39].

EREC quantifies the proportion of refractive error cases adequately corrected to normal vision thresholds (≥6/12).REC measures the broader provision of refractive correction regardless of optical adequacy. The definition of eREC (%) was calculated as: eREC (%) = ((met need)/(total need)) × 100. Refractive Error Coverage (REC) (%) was calculated as: REC (%) = (met need + under-met need)/(total need) × 100 [40]. Participants who wore spectacles and had distance VA worse than 6/12 in either eye without correction but achieved 6/12 or better with their own spectacles were categorized as ‘met need’. ‘Under-met need’ was defined as PVA worse than 6/12 with their present spectacles and could achieve 6/12 or better with best correction. ‘Unmet need’ was defined as the participants who had VA worse than 6/12 in either eye without correction and could achieve 6/12 or better with correction but did not wear spectacles. The sum of ‘met need’, ‘unmet need’, and ‘under-met need’ was considered as ‘total need’ (total prevalence of refractive error) [35], which represents the prevalence of need for spectacles. Need for Spectacles was defined as uncorrected visual acuity (UCVA) worse than 6/12 in either eye, with improvement to ≥6/12 in the same eye after refraction. This criterion aligns with WHO recommendations for addressing avoidable visual impairment in children and prior studies on refractive error coverage [41–43].

### Statistical analysis

Clinical examination forms were verified at least twice to evaluate integrity and precision before entering the database. Data were analyzed from November 1, 2023, through April 3, 2024, using SPSS statistical software for Windows, version 22 (SPSS, Chicago, IL, United States). Mean (± standard deviation), frequencies, and percentages were used to summarize the characteristics of the research subjects as appropriate. The prevalence of URE, VI and need for spectacles were the proportion of the total number of individuals who had uncorrected refraction, visual impairment and need for spectacles. The polynomial linear correlation in one-way ANOVA was used to test the trend of the prevalence of URE, VI and Need for glasses based on educational level and age category.

The association between URE, VI, need for spectacles and risk factors such as age, gender, educational stage, various types of refractive errors and anisometropia were assessed using multiple logistic regression analysis. The adjusted odds ratio (aOR) and 95% confidence intervals (CI) for the associated factors were calculated to describe the strength of association. Univariate and multivariate logistic regression models were constructed to determine the association between owning spectacles and characteristics among participants with the need for spectacles. All variables for multivariate logistic regression analysis were examined for multicollinearity. When height, weight, and BMI coexist, the variance in inflation factors for these three factors were all greater than 10. Therefore, weight was not included in the multiple regression analysis. When age stage and educational stage coexist, the variance inflation factors of both are greater than 5 in all four models. Because the education stage and age stage are highly correlated (Pearson correlation coefficient is 0.896 among the total participants), and the predictive ability of age stage is slightly higher than that of education stage in the four multiple regression models, age stage is used instead of education stage. All statistical tests were two-sided. The value of P < 0.05 was considered statistically significant.

## Results

### Characteristics of the study population

Of the 9,864 eligible participants in NSES, 9,438 (96.7%) completed the examination with complete refractive error data that were included in the analysis, with ages ranging from 7 to 19 years. Of the 9,438 participants, 46.7% (n = 4,412) were female. Mean SE was −2.53 ± 2.38 D (range −13.75 D to + 6.88 D). Four hundred and twenty-six students were excluded due to missing data. The total prevalence of myopia, hyperopia, astigmatism and anisometropia were 79.4% (95% CI: 78.6–80.2; n = 7,497), 2.7% (95% CI: 2.4–3.1; n = 258), 32.6% (95% CI: 31.7–33.5; n = 3,077) and 25.6% (95% CI: 24.7–26.5; n = 2,419), respectively. The prevalence of myopia, astigmatism and anisometropia showed an increase in trend with the educational stage and the age stage (all, p < 0.001; Table 1). The prevalence of hyperopia showed a decreased trend with the educational stage and the age stage (p < 0.001; Table 1).

**Table 1:** Educational stage- and Age-Specific Prevalence of Myopia, Hyperopia, Astigmatism, Anisometropia, URE, VI, need for spectacles, REC and eREC.

Characteristic		Educational stage, % (95% CI)				Age group, % (95% CI)							
	Total	Primary school	Junior high school	Senior high school	P value	7-8 y	9-10 y	11-12 y	13-14 y	15-16 y	17-18y	19y	P value
Myopia	79.4 (78.6-80.2)	65.4 (64.1-66.8)	92.7 (91.6-93.7)	95.6 (94.8-96.5)	<0.001*	38.1 (35.0-41.2)	57.0 (54.5-59.4)	79.4 (77.4-81.4)	89.0 (87.5-90.6)	94.1 (92.9-95.2)	95.1 (94-96.2)	96.6 (95.2-98.1)	<0.001*
Hyperopia	2.7 (2.4-3.1)	4.5 (3.9-5.0)	1.1 (0.7-1.6)	0.7 (0.4-1.1)	<0.001*	9.7 (7.8-11.6)	5.4 (4.2-6.5)	1.8 (1.1-2.5)	1.5 (0.9-2.1)	0.9 (0.5-1.4)	0.7 (0.3-1.1)	0.8 (0.1-1.5)	<0.001*
Astigmatism	32.6 (31.7-33.5)	23.6 (22.4-24.8)	37.4 (35.4-39.3)	47.1 (45.0-49.2)	<0.001*	17.1 (14.7-19.6)	18.2 (16.3-20.1)	29.1 (26.8-31.3)	32.6 (30.3-34.9)	40.7 (38.3-43.1)	46.0 (43.5-48.5)	46.5 (42.6-50.4)	<0.001*
Anisometropia	25.6 (24.7-26.5)	17.2 (16.1-18.3)	32.5 (30.6-34.4)	36.6 (34.6-38.6)	<0.001*	9.0 (7.2-10.8)	12.7 (11.0-14.4)	21.7 (19.7-23.7)	27.3 (25.1-29.5)	35.3 (33.0-37.7)	35.5 (33.1-37.9)	39.0 (35.1-42.8)	<0.001*
URE	15.7 (15.0-16.5)	14.1 (13.1-15.1)	21.2 (19.6-22.9)	13.3 (11.9-14.7)	=0.394	6.1 (4.6-7.7)	11.3 (9.7-12.9)	18.2 (16.3-20.1)	22.8 (20.7-24.8)	19.5 (17.6-21.5)	12.9 (11.2-14.6)	14.5 (11.8-17.3)	<0.001*
VI	4.9 (4.4-5.3)	5.2 (4.6-5.8)	5.4 (4.6-5.8)	3.6 (2.8-4.3)	0.003*	2.5 (1.5-3.5)	4.8 (3.8-5.9)	6.3 (5.1-7.5)	7.3 (6.0-8.5)	4.2 (3.2-5.1)	3.7 (2.7-4.6)	3.5 (2.1-5.0)	=0.685
need for spectacles	55.9 (54.9-56.9)	33.7 (32.4-35.1)	74.0 (72.3-75.8)	84.6 (83.1-86.1)	<0.001*	10.4 (8.4-12.3)	22.6 (20.5-24.7)	45.3 (42.8-47.7)	64.6 (62.3-67.5)	77.6 (75.6-79.7)	83.8 (81.9-85.6)	86.4 (83.7-89.1)	<0.001*
REC	78.6 (77.5-79.7)	64.6 (62.2-66.9)	79.8 (77.9-81.6)	89.7 (88.3-91.0)	<0.001*	48.0 (37.9-58.0)	56.0 (50.8-61.2)	66.8 (63.3-70.2)	72.9 (70.2-75.7)	82.6 (80.5-84.7)	89.6 (88.0-91.3)	89.3 (86.7-91.9)	<0.001*
e-REC	71.8 (70.6-73.1)	58.1 (55.7-60.5)	71.3 (69.2-73.4)	84.2 (82.6-85.9)	<0.001*	40.8 (30.9-50.7)	50.0 (44.7-55.3)	59.8 (56.2-63.5)	64.8 (61.9-67.7)	74.8 (72.4-77.3)	84.6 (82.7-86.6)	83.2 (80.0-86.3)	<0.001*

Analysis of polynomial linear correlation in one-way ANOVA was used for the trend test.

Abbreviations: URE, Uncorrected Refractive Error; VI, Visual Impairment, REC, Refractive Error Coverage; eREC, Effective Refractive Error Coverage.

*P < 0.05.

### Prevalence of Uncorrected refractive error (URE)

The total prevalence of URE in either eye was 15.7% (95% CI: 15.0–16.5; n = 1,485). In primary school, junior high school and senior high school, the prevalence of URE was 14.1% (95% CI: 13.1–15.1; n = 682), 21.2% (95% CI: 19.6–22.9; n = 507) and 13.3% (95% CI: 11.9–14.7; n = 296), respectively. URE prevalence did not vary significantly by educational stage (p = 0.394) but increased with age (p < 0.001). Compared with participants in the 7–8 age group, those in the older age groups had higher odds for URE, with the 13–14 age group having the highest odds (aOR: 3.92, 95% CI: 2.63–5.86, p < 0.001) for URE. (Table 1).

On multiple logistic regression analyses, participants with female sex (aOR: 1.24, 95% CI: 1.09–1.40, p < 0.001), hyperopia (aOR: 13.08, 95% CI: 7.67–22.31, p < 0.001), myopia (aOR: 18.65, 95% CI: 12.54–27.77, p < 0.001),anisometropia (aOR: 1.87, 95% CI: 1.64–2.12, p < 0.001) and living in the rural area (aOR: 1.34, 95% CI: 1.18–1.52, p < 0.001) had higher odds for URE. The prevalence of URE was lower among those participants with a higher BMI (aOR: 0.98, 95% CI: 0.96–0.99, p = 0.009) and participants who were wearing spectacles (aOR: 0.16, 95% CI: 0.14–0.19, p < 0.001) compared with those who did not (Table 2).

**Table 2:** Association of URE, VI, need for spectacles and Owning spectacles with characteristics (multivariable analysis).

Risk factor	Uncorrected Refractive Error (n = 1,485)			Visual Impairment (n = 459)			need for spectacles (n = 5,275)			Owning spectacles (n = 4,146)		
	Adjusted Odds ratio (95% CI)	n	P value	Adjusted Odds ratio (95% CI)	n	P value	Adjusted Odds ratio (95% CI)	n	P value	Adjusted Odds ratio (95% CI) value	n	P value
Age group												
7-8 y	1[Reference]	58		1[Reference]	24		1[Reference]	98		1[Reference]	47	
9-10 y	1.62 (1.16-2.25)	175	0.004*	1.70 (1.04-2.77)	75	0.035*	1.76 (1.34-2.31)	350	<0.001*	1.86 (1.31-2.65)	196	0.001*
11-12 y	2.59 (1.82-3.69)	284	<0.001*	2.50 (1.47-4.27)	98	0.001	2.92 (2.18-3.92)	707	<0.001*	3.10 (2.16-4.45)	472	<0.001*
13-14 y	3.92 (2.62-5.86)	363	<0.001*	4.30 (2.33-7.95)	116	<0.001*	4.90 (3.49-6.88)	1031	<0.001*	4.69 (3.16-6.96)	752	<0.001*
15-16 y	3.88 (2.49-6.06)	314	<0.001*	3.45 (1.71-6.93)	67	0.001*	7.32 (5.00-10.70)	1248	<0.001*	7.62 (4.99-11.64)	1031	<0.001*
17-18y	2.68 (1.66-4.32)	200	<0.001*	4.06 (1.91-8.63)	57	<0.001*	10.19 (6.77-15.34)	1300	<0.001*	12.34 (7.91-19.27)	1165	<0.001*
19y	3.12 (1.86-5.24)	91	<0.001*	3.98 (1.72-9.21)	22	0.001*	11.77 (7.44-18.64)	541	<0.001*	13.61 (8.44-21.95)	483	<0.001*
Height (cm)	1.00 (0.99-1.01)	NA	0.602	0.98 (0.98-1.00)	NA	0.069	1.02 (1.01-1.03)	NA	<0.001*	1.02 (1.02-1.03)	NA	<0.001*
BMI	0.98 (0.96-0.99)	NA	0.009*	0.97 (0.96-0.99)	NA	0.007*	0.97 (0.96-0.99)	NA	<0.001*	0.98 (0.97-0.99)	NA	0.005*
Sex												
Male	1[Reference]	731		1[Reference]	228		1[Reference]	2696		1[Reference]	2095	
Female	1.24 (1.09-1.40)	754	0.001*	1.20 (0.98-1.47)	231	0.075	1.58 (1.42-1.77)	2579	<0.001*	1.74 (1.56-1.94)	2051	<0.001*
Owning spectacles												
No	Reference	1129		Reference	389		NA	1129		NA	NA	
Yes	0.16 (0.14-0.19)	356	<0.001*	0.12 (0.09-0.16)	70	<0.001*	NA	4146	NA	NA	NA	
Hyperopia												
No	1[Reference]	1447		1[Reference]	456		1[Reference]	5190		1[Reference]	4090	
Yes	13.08 (7.67-22.31)	38	<0.001*	7.23 (1.60-32.61)	3	0.010*	16.56 (10.97-25.01)	85	<0.001*	16.13 (9.85-26.42)	56	<0.001*
Myopia												
No	1[Reference]	64		1[Reference]	7		1[Reference]	142		1[Reference]	90	
Yes	18.644 (12.51-27.77)	1421	<0.001*	53.04 (19.68-142.95)	452	<0.001*	28.88 (21.83-38.19)	5133	<0.001*	24.90 (17.46-35.50)	4056	<0.001*
Astigmatism												
No	1[Reference]	1031		1[Reference]	340		1[Reference]	2947		1[Reference]	2092	
Yes	1.09 (0.95-1.25)	454	0.218	1.09 (0.87-1.38)	119	0.444	2.50 (2.22-2.82)	2328	<0.001*	3.19 (2.84-3.55)	2054	<0.001*
Anisometropia												
No	1[Reference]	912		1[Reference]	381		1[Reference]	3530		1[Reference]	2850	
Yes	1.87 (1.64-2.12)	573	<0.001*	0.44 (0.34-0.57)	78	<0.001*	1.37 (1.21-1.55)	1745	<0.001*	0.78 (0.70-0.88)	1296	<0.001*
Urban-rural differences												
Urban	1[Reference]	590		1[Reference]	171		1[Reference]	2228		1[Reference]	1783	
rural	1.34 (1.18-1.52)	895	<0.001*	1.53 (1.25-1.87)	288	<0.001*	1.10 (0.98-1.22)	3047	0.101	0.89 (0.80-0.99)	2363	0.035*

Abbreviations: CI=95% confidence interval; NA=not applicable.

*P < 0.05.

### Prevalence of Visual Impairment (VI)

The total prevalence of VI was 4.9% (95% CI: 4.9–5.3; n = 459). In primary school, junior high school and senior high school, the prevalence of VI was 5.2% (95% CI: 4.6–5.8; n = 250),5.4% (95% CI: 4.6–5.8; n = 130) and 3.6% (95% CI: 2.8–4.3; n = 79), respectively. The prevalence of VI showed a decreased trend with the increase of educational stage but showed no trend with the increase of age stage (Table 1).

On multiple logistic regression analyses, participants with female sex (aOR: 1.20, 95% CI: 0.98–1.47, P < 0.001), hyperopia (aOR:7.23, 95% CI: 1.60–32.61, P < 0.001), myopia (aOR: 53.04, 95% CI: 19.68−142.95, P < 0.001), and living in the rural area (aOR: 1.53, 95% CI: 1.25–1.87, P < 0.001) had higher odds for VI. Compared with participants in the 7−8 age group, those in the older age groups had higher odds for VI, with the 13−14 age group having the highest odds (aOR: 4.30, 95% CI: 2.33–7.95 P < 0.001) for VI. The prevalence of VI. was lower among those participants with a higher BMI (aOR: 0.97, 95% CI: 0.96–0.99, P = 0.007), wearing spectacles (aOR: 0.12, 95% CI: 0.09–0.16, P < 0.001) and had anisometropia (aOR: 0.44, 95% CI: 0.34–0.57, P < 0.001) compared with those who did not (Table 2).

In addition, 51.2% of participants with inconsistent VI diagnoses between eyes had anisometropia, which was much higher than the prevalence of anisometropia in patients with consistent VI diagnoses between eyes (22.5%) (x^2 ^= 393.9, p < 0.001).

### Prevalence of need for spectacles

The total prevalence of need for spectacles was 55.9% (95% CI: 54.9–56.9; n = 5,275). In primary school, junior high school and senior high school, the prevalence of need for spectacles was 33.7% (95% CI: 32.4–35.1; n = 1,628), 74.0% (95% CI: 72.3–75.8; n = 1,769) and 84.6% (95% CI: 83.1–86.1; n = 1,878), respectively. The prevalence of need for spectacles showed an increase trend with the increase of educational stage and the age stage (Table 1).

On multiple logistic regression analyses, higher prevalence of need for spectacles was associated with those participants with female sex (aOR: 1.58, 95% CI: 1.42–1.77, p < 0.001), hyperopia (aOR: 16.56, 95% CI: 10.97–25.01 p < 0.001), myopia (aOR: 28.88, 95% CI: 21.83–38.19, p < 0.001), astigmatism (aOR:2.50, 95% CI: 2.22–2.82, P < 0.001), anisometropia (aOR: 1.37, 95% CI: 1.21–1.55, p < 0.001), lower BMI (aOR: 0.97, 95% CI: 0.96–0.99, p < 0.001) and higher height (aOR: 1.02, 95% CI: 1.01–1.03, p < 0.001). Compared with participants in the 7–8 age group, participants in older age groups have a higher odd for need for spectacles, with the 19-age group having the highest odds (aOR: 11.77, 95% CI:7.43–18.63, p < 0.001) (Table 2).

### Refractive Error Cvoverage (REC) and Effective Refractive Error Coverage (eREC)

The total prevalence of REC and e-REC were 78.6% (95% CI: 77.5–79.7; n = 4,146) and 71.8% (95% CI: 70.6–73.1; n = 3,790). In primary school, junior high school and senior high school, the prevalence of REC was 64.6% (95% CI: 62.2–66.9; n = 1,051), 79.8% (95% CI: 77.9–81.6; n = 1,411) and 89.7% (95% CI: 88.3–91.0; n = 1,684) and the prevalence of e-REC was 58.1% (95% CI: 55.7–60.5; n = 946), 71.3% (95% CI: 69.2–73.4; n = 1,262) and 84.2% (95% CI: 82.6–85.9; n = 1,582). The prevalence of REC and e-REC showed an increase trend of change with the educational stage and the age stage (all, p < 0.001; Table 1).

As shown in Table 3, multiple logistic regression analysis revealed that spectacle ownership among participants needing spectacles was significantly associated with female sex (aOR: 1.47, 95% CI: 1.27–1.71, p < 0.001), astigmatism (aOR:3.18, 95% CI: 2.71–3.73, P < 0.001) and higher height (aOR: 1.02, 95% CI: 1.01–1.03, p < 0.001). Compared with participants in the 7–8 age group, participants in older age groups have a higher odd for need for spectacles, with the 19-age group having the highest odds (aOR: 5.19, 95% CI:2.69–10.01, p < 0.001). The prevalence of owning spectacles was lower among those participants with anisometropia (aOR: 0.43, 95% CI: 0.37–0.50, p < 0.001) and participants who living in the rural area (aOR: 0.73, 95% CI: 0.63–0.84, p < 0.001).

**Table 3:** Univariate and multivariate logistic regression analysis of characteristics for Owning spectacles (n = 4,146) among participants with the need for spectacles (n = 5,275).

Characteristic	Univariate analysis			Multivariate analysis		
	Crude Odds ratio	95% CI	P value	Adjusted Odds ratio	95% CI	P value
Age group						
7-8 y	1[Reference]			1[Reference]		
9-10 y	1.38	0.88-2.16	0.159	1.29	0.80-2.10	0.297
11-12 y	2.18	1.42-3.34	<0.001*	1.60	0.97-2.63	0.064
13-14 y	2.93	1.92-4.45	<0.001*	1.79	1.05-3.08	0.034*
15-16 y	5.16	3.38-7.87	<0.001*	2.89	1.61-5.16	<0.001*
17-18y	9.36	6.06-14.46	<0.001*	4.98	2.70-9.19	<0.001*
19y	9.04	5.59-14.62	<0.001*	5.19	2.69-10.01	<0.001*
Educational stage						
Primary school	1[Reference]			NA	NA	NA
middle school	2.16	1.86-2.52	<0.001*	NA	NA	NA
high school	4.77	3.98-5.71	<0.001*	NA	NA	NA
Height (cm)	1.04	1.04-1.05	<0.001*	1.02	1.01-1.03	<0.001*
Weight (kg)	1.03	1.03-1.04	<0.001*	NA	NA	NA
BMI	1.03	1.03-1.04	<0.001*	1.00	0.98-1.02	0.957
Sex						
Male	1[Reference]			1[Reference]		
Female	1.11	0.98–1.27	0.107	1.47	1.27–1.71	<0.001*
Hyperopia						
No	1[Reference]			1[Reference]		
Yes	0.52	0.33–0.82	0.005	1.56	0.72-3.37	0.257
Myopia						
No	1[Reference]			1[Reference]		
Yes	2.18	1.54–3.08	<0.001*	1.54	0.84-2.83	0.16
Astigmatism						
No	1[Reference]			1[Reference]		
Yes	3.06	2.64–3.56	<0.001*	3.18	2.71–3.73	<0.001*
Anisometropia						
No	1[Reference]			1[Reference]		
Yes	0.63	0.55–0.73	<0.001*	0.43	0.37–0.50	<0.001*
Urban-rural differences						
Urban	1[Reference]			1[Reference]		
rural	0.89	0.78–1.02	0.083	0.73	0.63–0.84	<0.001*

Abbreviations: CI = 95% confidence interval; NA=not applicable.

* p < 0.05.

## Discussion

The current study showed that although the prevalence of VI among children and adolescents in eastern China was relatively low, we observed a high prevalence of URE and need for spectacles. Children and adolescents account for 18.16% of the total population in China [44], and they are more vulnerable group because URE may lead to poor adaptation and educational failure in children and adolescents [45]. Despite the potential magnitude of this issue, data on URE、need for spectacles, REC and eREC in general Chinese children and adolescents population are limited.

Prevalence estimates of need for spectacles and URE from the present study need to be interpreted cautiously. Previous studies [33,46] calculated the prevalence of the need for spectacles among populations who failed vision screening.

Consistent with other studies [40,47,48], the current study estimates the prevalence of need for spectacles in the entire sample representing the total prevalence of refractive error requiring optical correction. The current study showed that the prevalence of need for spectacles was related to older age, female gender, higher height and lower BMI, various types of refractive errors, and anisometropia, with myopia being the most important risk factor. Due to differences in definitions and age ranges, the prevalence of need for spectacles in different studies cannot be directly compared. In northwestern China, the prevalence of need for spectacles for senior high school students grades one and two defined as uncorrected VA ≤ 6/12 in the better eye which could be improved to >6/12 with refraction was 75% [48]. Similarly, the prevalence of need for spectacles in children and adolescents was 55.9%, and the prevalence among senior high school students was as high as 84.6% in the current study, indicating the high refractive correction needs of Chinese school-age children and adolescents. This was due to the high prevalence of myopia, astigmatism, and anisometropia among children and adolescents in eastern China worldwide [37,49,50].

In alignment with epidemiological studies on visual function in children and adolescents [32–34,51], both the definition of URE and need for spectacles considered both eyes to avoid missing unilateral visual dysfunction in this study. Consistent with most studies [15,52–55], VI was defined as PVA < 6/12 in the better eye, resulting in an overall prevalence of 4.9% among children and adolescents in eastern China, which is lower than the prevalence of 7.34% with the same definition globally [30]. In the current study, anisometropia was shown to be a protective factor for VI. This was because in the current study, the prevalence of anisometropia in participants with inconsistent VI diagnoses between eyes were much higher than that in participants with consistent VI diagnoses between eyes (51.2% vs 22.5%, p < 0.001), and these participants were not considered to have VI when diagnosed based on the better eye. In previous epidemiological studies on VI, URE. and need for spectacles, anisometropia was rarely mentioned. In the study by Mayro et al.[13] on the prevalence of URE among school-aged children in the School District of Philadelphia, the prevalence of anisometropia was 4.5%, but there was no further research on the impact of anisometropia on URE. In the study by Sherwin et al.[56] on URE among older British adults, the relationship between myopia, hyperopia, astigmatism and URE has been established, but the influence of anisometropia has not been taken into account. In the current research, the binocular condition was considered in both URE and the need for spectacles and anisometropia has been proven to be an important risk factor for URE and the need for spectacles, suggesting that in epidemiological studies of visual function, anisometropia was an important factor that should not be ignored, regardless of whether binocular conditions were considered or not.

The prevalence of URE in children and adolescents in eastern China was 15.7%, which was higher than that in Maharashtra, India (12.04%) [57] and California, USA (7.60%) [58]. Cao et al.[14] summarized the prevalence and causes of URE in children from the Global Burden of Disease sub-regional studies, showed that myopia and astigmatism are the main causes of URE worldwide due to the high prevalence. However, the current study confirms that the impact of different types of refractive errors on URE or VI cannot be determined solely by the prevalence of refractive errors. Various types of refractive errors (myopia, hyperopia, astigmatism) resulting in blurred retinal images [9] led them being risk factors for need for spectacles in the current research. Unlike need for spectacles, PVA was used when evaluating URE and VI. Due to the differences in research methods, demographic characteristics, living environments, socioeconomic status, education level, access to health insurance, ethnicity and individual factors (including psychological factors), there are differences in the perception of whether individuals with one or more kinds of refractive errors choose to undergo adequate refractive correction when experiencing URE or VI [47,59]. This kind of difference may be more complex in children and adolescents. Consistent with expectations, myopia and hyperopia are factors affecting URE and VI, with myopia being the most significant factor. Although there was also a high prevalence of astigmatism, astigmatism was confirmed not to be a risk factor for URE or VI in Children and adolescents in eastern China. Astigmatism often induces distinct symptoms such as blurred vision at all distances, glare, and eye strain [18]. These symptoms are more perceptible compared to spherical refractive errors [19]. Correction of astigmatism provides immediate and significant improvements in contrast sensitivity and functional vision [18], thereby reinforcing adherence to spectacle use. In contrast, parents of children with mild myopia or hyperopia may underestimate the urgency of refractive correction, leading to delayed adoption of spectacles [14]. Our supplementary analysis further supports this observation, demonstrating that astigmatism independently increased the likelihood of spectacle ownership by 3.18-fold among those requiring correction (adjusted OR: 3.18, 95% CI: 2.71–3.73; P < 0.001). In both the current study and previous literature reviews [56,60,61], wearing spectacles was the most important protective factor for URE and VI.

In the current study, although the prevalence of need for spectacles increases with age or educational stage, the prevalence of URE and VI were highest in 13–14 age group (junior high school in educational stage). This finding is consistent with Ma et al.[62], mainly due to the rapid growth of refractive errors and the lack of corrective spectacles in junior high school. Children and adolescents in the current study population receive at least one vision examination organized by the education department every year. There may be problems in the transition from school-based examinations to further medical institution examinations. Insufficient access to healthcare is one of the reasons for the lack of follow-up healthcare [63]. For policymakers, it is important to increase the intensity of screening in this educational stage and avoid insufficient opportunities for transitioning from school screening to subsequent medical care. Our data shows that girls are more likely to suffer from URE or VI than boys and also carry a significantly higher burden of need for spectacles than boys. This was consistent with previous research [62,64,65] and may be due to the additional near work and academic pressure and less time spent outdoors that girls face, which can lead to a higher risk of myopia.

The eREC was an important monitoring indicator for refractive error services. Bourne et al [66] reported that the prevalence of eREC varied across the regions. The high-income regions had the highest prevalence of eREC (79.7%). In Southeast Asia, east Asia, and Oceania, the prevalence of eREC was 40.0%, and in sub-Saharan Africa (5.7%) had the lowest prevalence of eREC. However, data on eREC in the age group of 10 to 29 was missing in their study. There is not much data on children and adolescents with eREC. Among a relatively young population aged 15–50 in Nepal [40], the prevalence of eREC and REC were 31.3% and 34.8%.Our data shows that the prevalence of eREC and REC among children and adolescents in eastern China were 71.8% and 78.6%, respectively. In a study involving seven provinces in China [62], the prevalence of REC increased from 13.0% at age 6 to 63.9% at age 17, with a total population prevalence of 35.9%. Our data shows the prevalence of REC increased from 52.6% in the 7–9 age group to 88.1% in the 16–19 age group. These reflect the high proportion of children and adolescents in eastern China who need refractive error services and have received them. However, it is worth noting that, in primary school, although the prevalence of children who need for spectacles (33.7%) was the lowest during the learning stage, the prevalence of eREC (58.1%) and REC (64.6%) were also the lowest. This may require policy managers to emphasize early diagnosis and intervention for children with refractive errors during this stage, especially clear guidance on methods suitable for preventing and controlling myopia, as myopia is rapidly increasing during primary school [67]. It is noteworthy that, as shown in Table 2, despite similar refractive correction needs between participants living in rural areas and those in urban areas. After adjustment for other characteristics, the results suggested that compared with living in urban areas, participants living in rural areas were 1.34 and 1.53 times more likely to suffer from URE (aOR: 1.34, 95% CI: 1.18–1.52, P < 0.001) and VI (aOR: 1.53, 95% CI: 1.25–1.87, P < 0.001), respectively.

This disparity arises because rural children and adolescents requiring spectacles had significantly lower rates of spectacle ownership compared to their urban counterparts (adjusted OR: 0.73, 95% CI: 0.63–0.85; P < 0.001). This suggests that additional attention should be given to children and adolescents in rural areas who require refractive correction. Eliminating common misunderstandings among parents about vision care is also important [68].

The strengths of this study include a large population representing the Children and adolescents in ordinary cities in eastern China, making the current results more universal. Due to its school-based nature, this study has a high response rate. More importantly, the current study provides evidence that anisometropia needs to be considered in epidemiological surveys related to URE, VI, and need for spectacles, which provides a reference for future research in terms of methodology. In the current study, anisometropia was studied together with various types of refractive errors and age factors, avoiding the guilt-by-association effect caused by potential correlations between them [17]. In addition, our results are valuable to China’s policy planners, as they provide key baseline information for achieving the WHO-approved goal of increasing the effective coverage of refractive errors by 40 percentage points by 2030 [69].

There were several limitations associated with our study. Orthokeratology and other contact lenses are important parts of refractive correction for children and adolescents in China [70]. Due to the considerations of PVA, to better estimate the prevalence of URE and VI in children and adolescents, the current study did not exclude participants who use orthokeratology and other types of contact lenses. Ocular and optical changes that occur with contact lenses will affect the results of optometry measurement [71]. It was more appropriate to use parameters before refractive correction for participants who use orthokeratology or other contact lenses. Second, there are racial differences in the correction of refractive errors [72] and these associations from a Han-dominated population may not be generalizable to other ethnic groups. Finally, further studies are needed to elucidate the potential causes of URE and VI. In addition to the factors included in the current study, it may also be necessary to include factors such as living environment, socioeconomic status, academic pressure, access to health insurance, race, education and awareness of eye care needs.

## Conclusion

This study showed that although the prevalence of VI among children and adolescents in eastern China was low, the prevalence of URE and the need for spectacles was high. The prevalence of URE and VI were highest in junior high school, while the prevalence of e-REC and REC were lowest in primary school. The impact of different types of refractive errors and anisometropia on URE or VI cannot be determined solely by the prevalence. Myopia is the most important risk factor for URE, VI, and need for spectacles, and the impact of anisometropia on URE, VI, and need for spectacles cannot be ignored. Further research on adjusting intervention strategies is needed to eliminate preventable visual impairments.

## Supporting information

S1 Table Univariate and multivariate logistic regression analysis of risk indicators for wearing spectacles among participants with the need for spectacles (n = 5,275) This is the S1 Table legend. (DOCX)
