# Supplementary material for: Uncorrected refractive errors, visual impairment and need for spectacles among children and adolescents in eastern, China
Source: PLoS One. 2025 Sep 16;20(9):e0332142. doi: 10.1371/journal.pone.0332142 (PMC12440161; doi:10.1371/journal.pone.0332142)
Supplement: S1 Table — (DOCX) [file pone.0332142.s001.docx]

**S1 Table**

**Univariate and multivariate logistic regression analysis of risk indicators for wearing spectacles among participants with the need for spectacles (n = 5,275)**

|  | **Univariate analysis** | | | **Multivariate analysis** | | |
| --- | --- | --- | --- | --- | --- | --- |
| **Characteristic** | **Crude Odds ratio** | **95% CI** | **p value** | **Adjusted Odds ratio** | **95% CI** | **p value** |
| Age | 1.25 | 1.22–1.28 | <0.001* | 1.29 | 1.26–1.33 | <0.001* |
| Sex | | | | | | |
| Male | Reference |  |  | Reference |  |  |
| Female | 1.11 | 0.98–1.27 | 0.107 | 1.31 | 1.13–1.51 | <0.001 |
| Myopia | | | | | | |
| Yes | 2.18 | 1.54–3.08 | <0.001* | NA | NA | NA |
| No | Reference |  |  | NA |  |  |
| Hyperopia | | | | | | |
| Yes | 0.52 | 0.33–0.82 | 0.005* | NA | NA | NA |
| No | Reference |  |  | NA |  |  |
| Astigmatism | | | | | | |
| Yes | 3.47 | 2.80–4.29 | <0.001* | 3.68 | 2.94–4.60 | <0.001* |
| No | Reference |  |  | Reference |  |  |
| Anisometropia | | | | | | |
| Yes | 0.63 | 0.55–0.73 | <0.001* | 0.44 | 0.38–0.52 | <0.001* |
| No | Reference |  |  | Reference |  |  |
| Axis position | | | | | | |
| With-the-rule | Reference |  |  | Reference |  |  |
| Against-the-rule | 0.63 | 0.43–0.92 | 0.015* | 0.65 | 0.43–0.97 | 0.036* |
| Oblique | 0.75 | 0.64–0.87 | <0.001* | 0.81 | 0.68–0.96 | 0.012* |

Abbreviations: CI = 95% confidence interval; NA = not applicable

^*^p < 0.05
